# Lysophosphatidic acid receptor mRNA levels in heart and white adipose tissue are associated with obesity in mice and humans

**DOI:** 10.1371/journal.pone.0189402

**Published:** 2017-12-13

**Authors:** Amy Brown, Intekhab Hossain, Lester J. Perez, Carine Nzirorera, Kathleen Tozer, Kenneth D’Souza, Purvi C. Trivedi, Christie Aguiar, Alexandra M. Yip, Jennifer Shea, Keith R. Brunt, Jean-Francois Legare, Ansar Hassan, Thomas Pulinilkunnil, Petra C. Kienesberger

**Affiliations:** 1 Department of Biochemistry and Molecular Biology, Faculty of Medicine, Dalhousie University, Dalhousie Medicine New Brunswick, Saint John, New Brunswick, Canada; 2 Cardiovascular Research New Brunswick, Saint John Regional Hospital, Saint John, New Brunswick, Canada; 3 Department of Pathology, Saint John Regional Hospital, Saint John, New Brunswick, Canada; 4 Department of Pharmacology, Faculty of Medicine, Dalhousie University, Dalhousie Medicine New Brunswick, Saint John, New Brunswick, Canada; 5 Department of Cardiac Surgery, New Brunswick Heart Centre, Saint John, New Brunswick, Canada; State University of Rio de Janeiro, BRAZIL

## Abstract

**Background:**

Lysophosphatidic acid (LPA) receptor signaling has been implicated in cardiovascular and obesity-related metabolic disease. However, the distribution and regulation of LPA receptors in the myocardium and adipose tissue remain unclear.

**Objectives:**

This study aimed to characterize the mRNA expression of LPA receptors (LPA1-6) in the murine and human myocardium and adipose tissue, and its regulation in response to obesity.

**Methods:**

LPA receptor mRNA levels were determined by qPCR in i) heart ventricles, isolated cardiomyocytes, and perigonadal adipose tissue from chow or high fat-high sucrose (HFHS)-fed male C57BL/6 mice, ii) 3T3-L1 adipocytes and HL-1 cardiomyocytes under conditions mimicking gluco/lipotoxicity, and iii) human atrial and subcutaneous adipose tissue from non-obese, pre-obese, and obese cardiac surgery patients.

**Results:**

LPA1-6 were expressed in myocardium and white adipose tissue from mice and humans, except for LPA3, which was undetectable in murine adipocytes and human adipose tissue. Obesity was associated with increased LPA4, LPA5 and/or LPA6 levels in mice ventricles and cardiomyocytes, HL-1 cells exposed to high palmitate, and human atrial tissue. LPA4 and LPA5 mRNA levels in human atrial tissue correlated with measures of obesity. LPA5 mRNA levels were increased in HFHS-fed mice and insulin resistant adipocytes, yet were reduced in adipose tissue from obese patients. LPA4, LPA5, and LPA6 mRNA levels in human adipose tissue were negatively associated with measures of obesity and cardiac surgery outcomes. This study suggests that obesity leads to marked changes in LPA receptor expression in the murine and human heart and white adipose tissue that may alter LPA receptor signaling during obesity.

## Introduction

Lysophosphatidic acid (LPA) is a small glycerophospholipid and bioactive molecule that is present in interstitial fluid and blood, with circulating LPA concentrations ranging from 0.1 μM to 10 μM in plasma and serum, respectively [1]. LPA exerts pleiotropic signaling roles in vertebrates that encompass the regulation of cell differentiation [2], migration [3], survival [4], and cytoskeletal reorganization [5, 6]. The majority of LPA is produced by the secreted enzyme autotaxin, a lysophospholipase D that hydrolyzes lysophosphatidylcholine (LPC) contained in lipoproteins [1, 7]. At present, six rhodopsin-like 7-transmembrane receptors (LPA1-6) have been identified that are coupled to one or more G_α_ protein [1, 8]. LPA receptors are categorized into two subfamilies, the endothelial differentiation, G-protein-coupled (EDG) family of LPA receptors (LPA1-3) and non-EDG family (LPA4-6) with similarity to purinergic receptors [8]. Although most LPA receptors are broadly expressed, they vary significantly in tissue distribution and relative abundance and appear to have both overlapping and distinct biological roles [5].

LPA receptor signaling has been implicated in cardiovascular disease [9–18]. LPA levels are elevated in atherosclerotic plaque and LPA receptor signaling may promote atherosclerosis development, inflammation, and thrombosis [14, 15]. Prior studies also showed that LPA levels increase with myocardial infarction [16, 17]. On the other hand, a recent study suggested that LPA1 and LPA3 signaling provide a protective effect against ischemia-reperfusion injury in rats and sequelae from myocardial infarction in mice [11, 13]. Bouchareb et al. [12] identified the autotaxin-LPA signaling axis as a major contributor to aortic valve stenosis as it promotes aortic valve inflammation and mineralization. Moreover, increased LPA signaling in cardiomyocytes was linked to cardiac hypertrophy and heart failure in mice [18]. Collectively, these studies suggest that LPA receptors play an important role in cardiovascular diseases including atherosclerotic disease, myocardial injury, aortic valve stenosis, cardiac hypertrophy, and heart failure. However, little is known about the regulation and significance of specific LPA receptors in the heart muscle, specifically cardiomyocytes.

LPA receptor signaling has also been implicated in the regulation of glucose homeostasis and obesity-related metabolic disease in mice and humans [19–23]. Studies using mice with whole body heterozygous autotaxin deficiency or adipose-specific autotaxin deficiency have suggested that a chronic reduction in autotaxin-LPA signaling protects against impaired glucose homeostasis during diet-induced obesity [19, 20]. Consistent with these findings in mouse models, most clinical studies have associated the autotaxin-LPA pathway with obesity, insulin resistance and impaired glucose homeostasis in humans [20–23]. What is lacking is a comparison of the receptor subtypes between a relevant human population and animal/cellular models of cardiovascular and metabolic disease to explore and validate drug targets.

We hypothesize that distinct LPA receptors are upregulated in heart and adipose tissue during obesity and that the expression of these LPA receptors is related to the metabolic and cardiovascular morbidity in individuals undergoing cardiac surgery. Here we show a detailed characterization of the mRNA expression patterns of known bona fide LPA receptors in the myocardium and adipose tissue of humans with an examination of regulation in response to obesity, and determine whether LPA receptor expression is correlated to metabolic parameters and outcomes after cardiac surgery. We also show LPA receptor expression in heart and adipose tissue from mice and cultured cardiomyocytes and adipocytes to determine whether LPA receptor regulation in response to obesogenic stimuli is comparable among humans and animal/cell models of metabolic and cardiovascular disease that are used for drug development and preclinical validation.

## Materials and methods

### Clinical sampling

**Fig 1:**
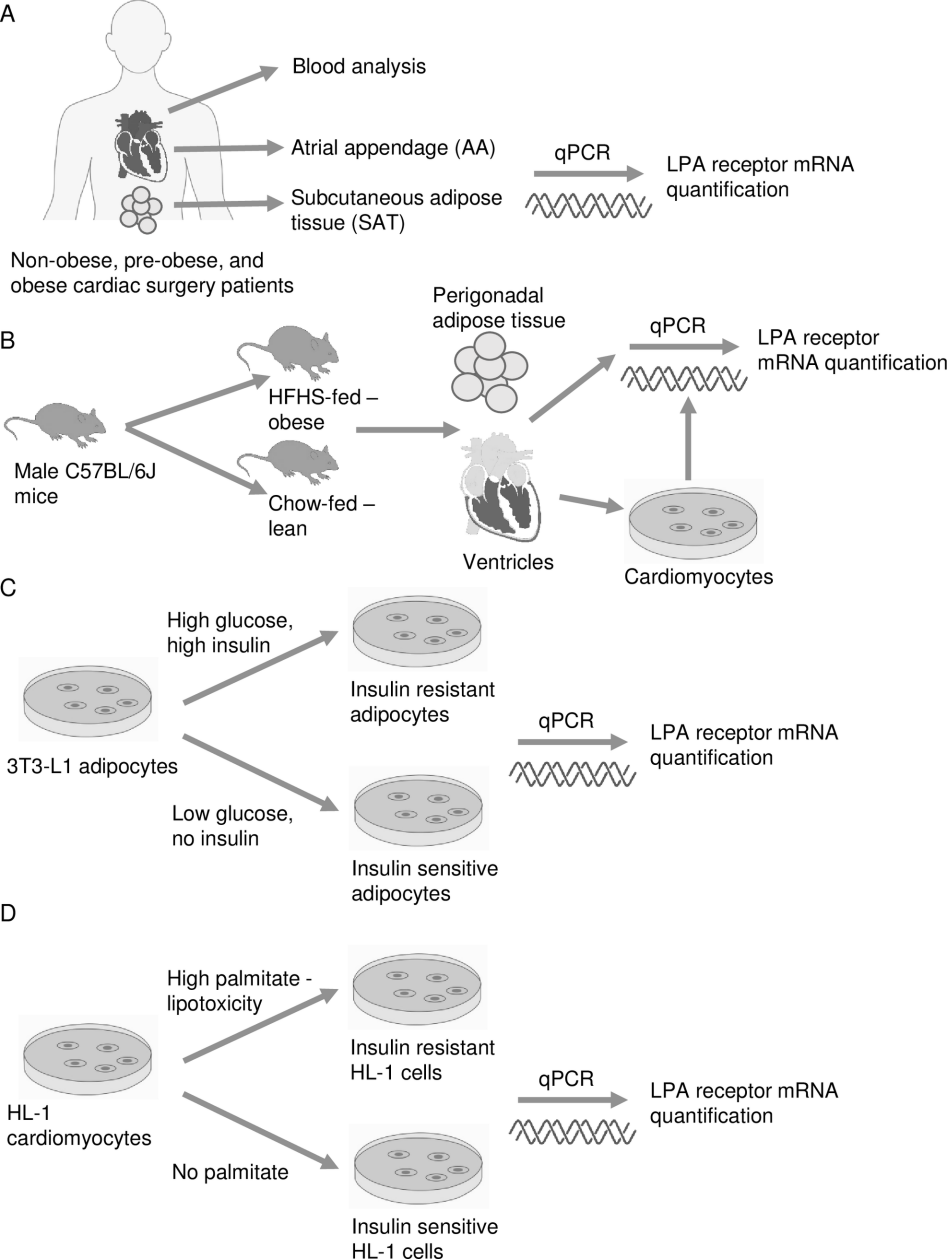
Experimental design and groups for LPA receptor expression analysis. Experimental layout of LPA receptor mRNA quantification in A) heart and adipose tissue from non-obese, pre-obese, and obese cardiac surgery patients, B) heart, adipose tissue, and cardiomyocytes from lean and obese mice, C) insulin sensitive and insulin resistant 3T3-L1 adipocytes, and D) insulin sensitive and insulin resistant HL-1 cardiomyocytes.

All protocols involving human subjects were approved by the Research Ethics Board of the Saint John Regional Hospital, NB, Canada (protocol #2014–2006). Human tissue samples were collected from patients undergoing elective, first-time cardiac surgery (coronary artery bypass graft and/or valve surgery) at the New Brunswick Heart Centre in Saint John, NB, Canada who provided consent to be enrolled in the study. Immediately before surgery, a venous blood sample was collected. During the surgery, samples of the right atrial appendage (AA) and thoracic subcutaneous adipose tissue (SAT) were excised, frozen in liquid nitrogen, and stored at -80°C until further processing for quantitative polymerase chain reaction (qPCR) analysis (Fig 1).

### Experimental models of obesity

All protocols involving mice were approved by the Dalhousie University Institutional Animal Care and Use Committee. Male C57BL/6J mice (The Jackson Laboratory) were housed on a 12 h light: 12 h dark cycle with ad libitum access to food and water. Eight week-old mice were randomly assigned to cohorts fed either chow (5001, Lab Diet) or high fat-high sucrose (HFHS) diet (12451, Research Diets)(Table 1) and fed for 16 weeks [24]. Hearts and perigonadal adipose tissue were collected from fed and 16 h-fasted mice following euthanasia by decapitation to examine the regulation of LPA receptor expression in response to chronic obesity and acute changes in nutritional status (Fig 1). For subsequent qPCR studies, heart ventricles and adipose tissue samples were snap-frozen in liquid nitrogen and stored at -80°C.

**Table 1:** Nutritional composition of research diets.

Nutrient class	Chow diet	HFHS diet
	(Lab Diet 5001)	(Research Diets 12451)
	***% kcal of total***	
Protein	30	20
Fat	13	45
Carbohydrate	57	35 (Sucrose: 17)
Kcal/g	4.1	4.7

### Primary mouse cardiomyocytes

Cardiomyocytes were isolated from chow and HFHS-fed C57BL/6J mice as previously described [25]. Excised hearts were immersed in ice-cold mouse heart perfusion buffer containing 113 mM NaCl, 14.7 mM KCl, 0.6 mM KH_2_PO_4_, 0.6 mM Na_2_HPO_4,_ 1.2 mM MgSO_4_, 12 mM NaHCO_3,_ 10 mM KHCO_3,_ 10 mM HEPES, 30 mM taurine, 10 mM BDM and 5.5 mM glucose, pH 7.4. Subsequently, hearts were retrogradely perfused via the aorta and subjected to collagenase (75 mg collagenase in 50 ml perfusion buffer at 37°C) digestion for 18 min in recirculating mode. Thereafter, ventricles were minced into small pieces and the mixture was strained (100 μm) to obtain a homogenous cell suspension. Ventricular myocytes were made calcium tolerant by exposing the cells to increasing concentrations of calcium (100, 400, and 900 μM). Viable cardiomyocytes were collected and processed for qPCR analysis (Fig 1).

### Adipocyte and HL-1 cell culture

3T3-L1 cells (ATCC) were cultured and differentiated to mature adipocytes as previously described [24]. Insulin resistance was induced by exposing adipocytes to media containing high glucose (25 mM) and high insulin (100 nM) concentrations for 24 h [24] (Fig 1). Insulin-sensitive control adipocytes were incubated with low glucose (6.1 mM) and in the absence of insulin [24]. Cells were collected for qPCR analysis as detailed previously [24]. To assess insulin signaling in 3T3-L1 adipocytes, cells were washed once in phosphate-buffered saline (PBS) and stimulated with 20 nM insulin from bovine pancreas (Sigma) in 1 ml DMEM-1X (Thermo Fisher Scientific) containing 1.1 g/L glucose for 15 min. Control cells were incubated in the absence of insulin. HL-1 cardiomyocytes (kindly provided by Dr. Claycomb [26]) were maintained in fibronectin–gelatine coated flasks containing Claycomb medium (Sigma) and supplemented with 10% fetal bovine serum, 2 mM L-glutamine and 0.1 mM norepinephrine. Cells were cultured at 37°C and 5% CO_2_. To induced insulin resistance, HL-1 cells were incubated with DMEM-1X supplemented with 5 mM glucose and 1.2 mM sodium palmitate for 18 h. Palmitate-containing media was prepared as described in Trivedi et. al. [27] with modifications. Briefly, a 120 mM stock solution of sodium palmitate (Sigma) was prepared in DMEM-1X. The stock solution was then added to DMEM-1X supplemented with 5 mM glucose and 2% (w/v) fatty acid-free BSA, pre-warmed at 50°C, and the mixture was incubated at 37°C for 30 min. The solution containing BSA-complexed palmitate was sterile-filtered and immediately used to incubate cells. Controls were cultured in the absence of palmitate. To examine insulin signaling, cells were washed with PBS and incubated in the absence or presence of 100 nM insulin for 15 min. To collect 3T3-L1 and HL-1 cells, cells were washed in PBS, scraped and pelleted through centrifugation at 10,000 x g for 10 min at 4°C. For subsequent immunoblotting analysis, cell pellets were lysed through sonication in lysis buffer (20 mM Tris-HCl pH 7.5, 5 mM EDTA, 10 mM Na_4_P_2_O_7_, 100 mM NaF, 1% NP-40) containing 2 mM sodium orthovanadate, 2 mM protease inhibitor cocktail (P8340, Sigma) and 100 μg/mL phosphatase inhibitor cocktail (524628, Calbiochem). Protein concentrations were quantified colorimetrically using a bicinchoninic acid (BCA) protein assay kit (Thermo Scientific) and BSA as standard.

### Immunoblot analysis

Cell lysates were subjected to SDS-PAGE and proteins were transferred onto a nitrocellulose membrane. Transferred proteins were visualized using a reversible protein stain (Memcode, Pierce, Thermo Fisher Scientific) and membranes were incubated overnight with the following primary antibodies: anti-pAkt Ser^473^ (9271, Cell Signaling) and anti-Akt (05–591, Millipore). Immunoblots were developed using the Western Lightning Plus-ECL enhanced chemiluminescence substrate (Perkin Elmer). Densitometric analysis was performed using Image lab software (Bio-Rad).

### Quantification of LPA receptor mRNA

LPA receptor mRNA levels in tissues and cells were determined using qPCR using validated optimal reference gene pairs as previously described [28]. Primer information for LPA receptor and reference genes is provided in Table 2. Ground tissue samples and harvested cells were homogenized in Ribozol (Amresco), RNA was isolated as per the manufacturer’s instructions, and RNA quality and quantity were examined using a QIAxcel Advanced System (Qiagen). cDNA was synthesized from 1 μg of RNA using qScript cDNA supermix (Quanta Biosciences) and cDNA samples were stored at −20°C until further use. qPCR analysis was performed in 96-well plates using PerfeCTa SYBR green Supermix Low ROX (Thermo Fisher Scientific) and a ViiA7 real-time PCR machine (Thermo Fisher Scientific) as detailed previously [28]. mRNA levels were quantified using gbase + software (Biogazelle) [28].

**Table 2:** Sequence information for primers employed in qPCR analysis.

Species	Gene	Forward (top) and reverse (bottom) primer sequence (5’ to 3’)
***Lysophosphatidic acid receptors***		
**Mouse**	*Lpar1*	CTATGTTCGCCAGAGGACTAT
		GCAATAACAAGACCAATCCCG
	*Lpar2*	CACACTCAGCCTAGTCAAGA
		GTACTTCTCCACAGCCAGAA
	*Lpar3*	ACCAACGTCTTATCTCCACAC
		CAGTTCAGGCCGTCCAGC
	*Lpar4*	AGGATGGAGTCGCTGTTTAAG
		CACCACCATTATTTGTTGTTTGATC
	*Lpar5*	CCTCAGACTAATTTCTCTTCCC
		GTATCTCGATAGTCAGGGCAC
	*Lpar6*	CTCCAATGGCTCCCAGTG
		GGATATCAGCCCAAGCACG
**Human**	*LPAR1*	GGCTATGTTCGCCAGAGGACTAT
		TCCAGGAGTCCAGCAGATGATAA
	*LPAR2*	CAGCCTGGTCAAGACTGTTGT
		TGCAGGACTCACAGCCTAAA
	*LPAR3*	ACGGTGATGACTGTCTTAGGG
		CACCTTTTCACATGCTGCAC
	*LPAR4*	AAAGATCATGTACCCAATCACCTT
		CTTAAACAGGGACTCCATTCTGAT
	*LPAR5*	ATGTTAGCCAACAGCTCCTCAACC
		GCCAGTGGTGCAGTGCGTAGTA
	*LPAR6*	GGTAAGCGTTAACAGCTCCCACT
		TTTGAGGACGCAGATGAAAATGT
***Reference genes***		
**Mouse**	*Rpl7*	ACGGTGGAGCCTTATGTGAC
		TCCGTCAGAGGGACTGTCTT
	*Rpl27*	AAGCCGTCATCGTGAAGAACA
		CTTGATCTTGGATCGCTTGGC
	*Rpl41*	GCCATGAGAGCGAAGTGG
		CTCCTGCAGGCGTCGTAG
	*Ppia*	GGGTTCCTCCTTTCACAGAA
		GATGCCAGGACCTGTATGCT
	*Hprtl*	CAGTCCCAGCGTCGTGATTA
		GGCCTCCCATCTCCTTCATG
	*Rn18s*	GGCCGTTCTTAGTTGGTGGAGCG
		CTGAACGCCACTTGTCCCTC
**Human**	*YWHAZ*	ACTTTTGGTACATTGTGGCTTCAA
		CCGCCAGGACAAACCAGTAT
	*PPIA*	AGACAAGGTCCCAAAGAC
		ACCACCCTGACACATAAA
	*ACTB*	ATGAAGATCAAGATCATTGCTCCTC
		ACATCTGCTGGAAGGTGGACA
	*SDHA*	TGGGAACAAGAGGGCATCTG
		CCACCACTGCATCAAATTCATG
	*HSCPB*	TCTGGGTATCGGAAAGCAAGCC
		GTGCACTTCCTCAGGCATCTTG

### Statistical analysis

Data are expressed as mean ± standard error of the mean (SEM) unless otherwise indicated. Statistical and Pearson’s correlation analyses were conducted using Prism software (GraphPad) and SAS statistical software version 9.4, respectively. Comparisons between two groups were performed using unpaired two-tailed Student’s t-test and comparisons between multiple groups were performed using two-way analysis of variance followed by a Tukey or Sidak’s post-hoc test or one-way ANOVA followed by a Tukey post-hoc test, as appropriate. *P* values of less than 0.05 were considered statistically significant.

## Results

### Patient characteristics and clinical outcomes

Patient characteristics and pre-operative parameters are summarized in Tables 3 and 4. Age at time of surgery ranged from 44 to 74 years and was similar between BMI groups. Patients with a BMI of 18.5–24.9 kg/m^2^ were considered as the non-obese control group (n = 6), patients with a BMI of 25.0–29.9 kg/m^2^ were classified as pre-obese (n = 6), and patients with a BMI of ≥30.0 kg/m^2^ were classified as obese (n = 18, spanning class I to class III obesity). Accordingly, the mean waist circumference was 86.1 cm, 103.3 cm, and 120.7 cm for non-obese, pre-obese, and obese patients, respectively. Current smokers comprised 33% of non-obese, 17% of pre-obese, and 0% of obese patients. Patients in the obese category had the highest rates of diabetes mellitus type 2 and hypercholesterolemia. Accordingly, HbA1c levels were elevated in obese compared to non-obese individuals, although other pre-operative metabolic parameters were similar across BMI groups. The majority of patients in each BMI category (100% of non-obese patients, 83% of pre-obese patients, and 94% of obese patients) were taking beta-blockers, thiazide diuretics, angiotensin-converting enzyme inhibitors and/or angiotensin receptor blockers for blood pressure management. Additionally, a comparable proportion of patients in each BMI group (67% of non-obese patients, 67% of pre-obese patients, and 72% of obese patients) were taking statins for lipid lowering. Parameters from the pre-op complete blood counts, blood chemistry, and liver panel were not significantly different between patients in different BMI categories. Pre-operative echocardiography and angiography showed no differences in heart function between groups. Pre-operatively, 17% of obese patients presented with a history of myocardial infarction and 61% with angina, compared to 0% and 83% of pre-obese patients, and 17% and 83% of non-obese patients, respectively. Heart failure in NYHA Class 3 and 4 categories was evident preoperatively in 17% of non-obese, 33% of pre-obese, and 61% of obese patients. Post-operative clinical parameters are summarized in Table 5. The post-operative length-of-stay was significantly longer for obese patients compared to pre-obese but not non-obese patients. Only 67% of obese patients were discharged directly back home compared to 83% and 100% of pre-obese and non-obese patients, respectively.

**Table 3:** Patient characteristics and metabolic parameters.

Parameter			Non-obese	Pre-obese	Obese	Statistical analysis^1^
			(BMI 18.5–24.9 kg/m^2^)	(BMI 25–29.9 kg/m^2^)	(BMI ≥30 kg/m^2^)	
			n = 6	n = 6	n = 18	
**Demographic data**						
	**Male/Female**		3/3	5/1	13/5	
	**Age (years)**		61.0 (11.0)	64.2 (8.2)	64.7 (5.3)	N v PO: ns
						N v O: ns
						PO v O: ns
	**Smoker (n)**		2 (33)	1 (17)	0 (0)	
**Medications**						
	**Blood pressure- lowering (n)**		6 (100)	5 (83)	17 (94)	
		**Beta blocker (n)**	3 (50)	2 (33)	11 (61)	
		**Diuretic (n)**	0 (0)	2 (33)	9 (50)	
		**ACE inhibitor (n)**	5 (83)	2 (33)	10 (56)	
		**Angiotensin Receptor Blocker (n)**	0 (0)	1 (17)	5 (28)	
		**Lipid-lowering (n)**	4 (67)	4 (67)	13 (72)	
**Parameters of obesity**						
	**BMI (kg/m**^**2**^**)**		22.3 (1.4)	28.4 (1.1)	36.6 (4.3)	N v PO: *
						N v O: ****
						PO v O: ***
	**Waist circumference (cm)**		86.1 (12.3)	103.4 (10.3)	120.7 (14.6)	N v PO: ns
						N v O: ****
						PO v O: *
	**Hip circumference (cm)**		99.0 (8.8)	101.9 (2.8)	120.9 (14.8)	N v PO: ns
						N v O: **
						PO v O: **
**Metabolic parameters**						
	**Hypertension (n)**		6 (100)	4 (67)	15 (83)	
	**Triacylglycerols fasting (mmol/L)**		1.07 (0.53)	1.41 (0.76)	1.75 (0.76)	N v PO: ns
						N v O: ns
						PO v O: ns
	**HDL cholesterol fasting (mmol/L)**		1.50 (0.42)	1.16 (0.24)	1.18 (0.38)	N v PO: ns
						N v O: ns
						PO v O: ns
	**Hypercholesterolemia (n)**		3 (50)	4 (67)	14 (78)	N v PO: ns
						N v O: ns
						PO v O: ns
	**Cholesterol fasting (mmol/L)**		4.28 (1.38)	4.22 (1.17)	3.90 (0.91)	N v PO: ns
						N v O: ns
						PO v O: ns
	**LDL cholesterol fasting (mmol/L)**		2.31 (1.08)	2.42 (0.95)	1.83 (0.77)	N v PO: ns
						N v O: ns
						PO v O: ns
	**Non-HDL cholesterol fasting (mmol/L)**		2.80 (1.11)	3.06 (1.21)	2.72 (0.76)	N v PO: ns
						N v O: ns
						PO v O: ns
	**Diabetes mellitus type 2 (n)**		0 (0)	2 (33)	6 (33)	
	**HbA1c (%)**		5.27 (0.23)	5.55 (0.43)	5.9 (0.56)	N v PO: ns
						N v O: *
						PO v O: ns
	**Glucose random blood (mmol/L)**		5.22 (0.73)	6.40 (2.79)	7.31 (2.39)	N v PO: ns
						N v O: ns
						PO v O: ns
	**Fasting Glucose (mmol/L)**		5.58 (0.73)	8 (4.05)	6.9 (1.62)	N v PO: ns
						N v O: ns
						PO v O: ns
	**Fasting Insulin (mU/L)**		19.5 (16.5)	29.6 (27.6)	21.1 (19.6)	N v PO: ns
						N v O: ns
						PO v O: ns
	**HOMA-IR**^**2**^ **(mg/dL)**		5.07 (4.99)	10.84 (9.45)	6.91 (6.96)	N v PO: ns
						N v O: ns
						PO v O: ns
	**hsCRP (mg/L)**		1.81 (2.15)	1.92 (1.58)	2.71 (1.76)	N v PO: ns
						N v O: ns
						PO v O: ns
	**Metabolically healthy**^**3**^ **(n)**		3 (50)	1 (17)	2 (33)	

Data are expressed as mean (standard deviation) for continuous variables and number (percent of population) for categorical values. Statistical analysis for categorical values was performed using a one-way ANOVA followed by a Tukey post hoc test.

^1^ N = non-obese, PO = pre-obese, O = obese, ns = non-significant, *P < 0.05, **P < 0.01, ***P < 0.001, ****P < 0.0001.

^2^ HOMA-IR = (glucose x insulin)/405.

^3^ Criteria based on blood pressure, triglycerides, HDL-C, glucose, HOMA-IR, and hsCRP as outlined in Wildman et al. [29].

**Table 4:** Pre-operative blood and cardiovascular parameters.

Parameter		Non-obese	Pre-obese	Obese	Statistical analysis^1^
		(BMI 18.5–24.9 kg/m^2^)	(BMI 25–29.9 kg/m^2^)	(BMI ≥30 kg/m^2^)	
		n = 6	n = 6	n = 18	
**Complete blood counts**					
	**Leukocytes (x10**^**9**^**/L)**	6.90 (1.63)	6.55 (1.33)	6.93 (1.49)	N v PO: ns
					N v O: ns
					PO v O: ns
	**Erythrocytes (x10**^**12**^**/L)**	4.08 (0.55)	4.01 (0.79)	4.39 (0.45)	N v PO: ns
					N v O: ns
					PO v O: ns
	**Hemoglobin (g/L)**	129 (20)	120 (25)	133 (15)	N v PO: ns
					N v O: ns
					PO v O: ns
	**Hematocrit (L/L)**	0.381 (0.056)	0.357 (0.076)	0.395 (0.042)	N v PO: ns
					N v O: ns
					PO v O: ns
	**Platelets (x10**^**9**^**/L)**	175 (12)	190 (32)	192 (39)	N v PO: ns
					N v O: ns
					PO v O: ns
	**Neutrophils (x10**^**9**^**/L)**	4.52 (1.10)	4.47 (1.23)	4.61 (1.25)	N v PO: ns
					N v O: ns
					PO v O: ns
	**Lymphocytes (x10**^**9**^**/L)**	1.57 (0.95)	1.53 (0.25)	1.46 (0.54)	N v PO: ns
					N v O: ns
					PO v O: ns
**Blood chemistry**					
	**Sodium (mmol/L)**	138.83 (4.67)	140.33 (2.94)	140.55 (2.28)	N v PO: ns
					N v O: ns
					PO v O: ns
	**Potassium (mmol/L)**	4.33 (0.36)	4.08 (0.17)	4.15 (0.33)	N v PO: ns
					N v O: ns
					PO v O: ns
	**Chloride (mmol/L)**	102 (6)	101 (3)	103 (3)	N v PO: ns
					N v O: ns
					PO v O: ns
	**Creatinine (mmol/L)**	90.5 (36.8)	87.7 (16.6)	84 (31.8)	N v PO: ns
					N v O: ns
					PO v O: ns
	**Urea (mmol/L)**	6.58 (2.35)	6.15 (1.38)	6.6 (2.37)	N v PO: ns
					N v O: ns
					PO v O: ns
	**Troponin T high-sensitive (ng/L)**	16.8 (12.4)	9.2 (2.5)	17.7 (24.4)	N v PO: ns
					N v O: ns
					PO v O: ns
**Liver panel**					
	**ALT (U/L)**	29.4 (23.1)	27.0 (18.9)	25.0 (9.6)	N v PO: ns
					N v O: ns
					PO v O: ns
	**AST (U/L)**	37.2 (30.0)	24.0 (17.1)	25.0 (8.7)	N v PO: ns
					N v O: ns
					PO v O: ns
**Echocardiography/angiography**					
	**Ejection fraction (%)**	65.3 (15.1)	59.2 (14.9)	59.9 (11.9)	N v PO: ns
					N v O: ns
					PO v O: ns
	**Left ventricular end diastolic pressure (mmHg)**	17.7 (9.0)	14.5 (4.4)	22.4 (14.2)	N v PO: ns
					N v O: ns
					PO v O: ns
**Complications**					
	**Myocardial Infarction (n)**	1 (17)	0 (0)	3 (17)	
	**Angina (n)**	5 (83)	5 (83)	11 (61)	
**NYHA Classification**					
	**1 (n)**	2 (33)	1 (17)	1 (6)	
	**2 (n)**	3 (50)	3 (50)	5 (28)	
	**3 (n)**	1 (17)	1 (17)	11 (61)	
	**4 (n)**	0	1 (17)	0 (0)	

Data are expressed as mean (standard deviation) for continuous variables and number (percent of population) for categorical values. Statistical analysis for categorical values was performed using a one-way ANOVA followed by a Tukey post hoc test.

^1^ N = non-obese, PO = pre-obese, O = obese, ns = non-significant.

**Table 5:** Post-operative parameters and outcomes.

Parameter		Non-obese	Pre-obese	Obese	Statistical analysis^1^
		(BMI 18.5–24.9 kg/m^2^)	(BMI 25–29.9 kg/m^2^)	(BMI ≥30 kg/m^2^)	
		n = 6	n = 6	n = 18	
**Blood chemistry**					
	**Post-OP Troponin (ng/L)**	977 (389)	704 (366)	1016 (946)	N v PO: ns
					N v O: ns
					PO v O: ns
**Post-OP complications**					
	**Length of stay post-surgery (days)**	6.83 (1.47)	5.50 (0.55)	9.60 (3.61)	N v PO: ns
					N v O: ns
					PO v O: *
	**Reoperation (n)**	0 (0)	0 (0)	0 (0)	
	**Infection (n)**	0 (0)	0 (0)	1 (6)	
	**Renal failure (n)**	0 (0)	0 (0)	4 (22)	
	**In-hospital mortality (n)**	0 (0)	0 (0)	1 (6)	
	**Home discharge (n)**	6 (100)	5 (83)	12 (67)	

Data are expressed as mean (standard deviation) for continuous variables and number (percent of population) for categorical values. Statistical analysis for categorical values was performed using a one-way ANOVA followed by a Tukey post hoc test.

^1^ N = non-obese, PO = pre-obese, O = obese. ns = non-significant, *P < 0.05.

### Diet-induced obesity leads to changes in the LPA receptor mRNA expression profile in the murine heart and cardiomyocytes

**Fig 2:**
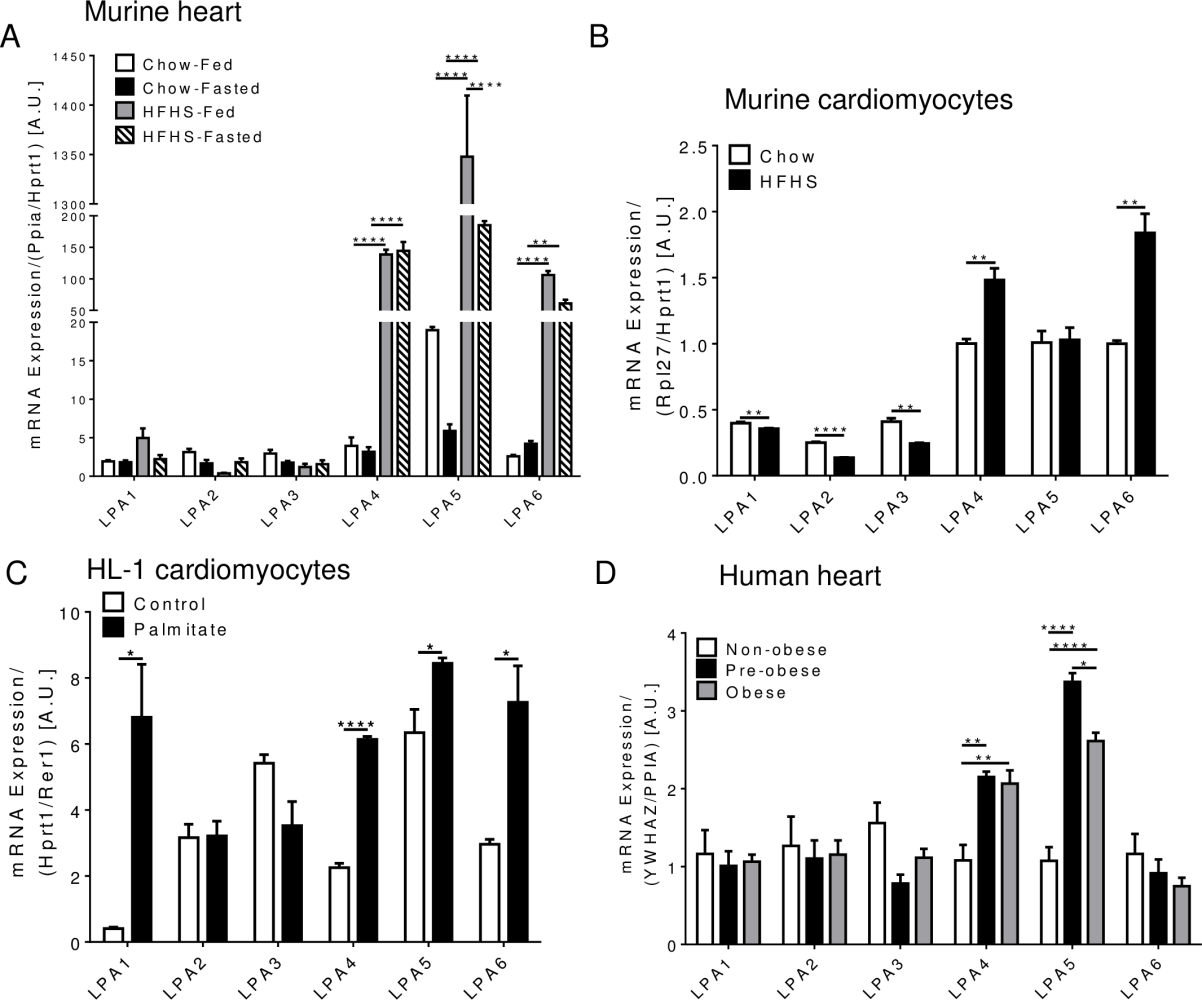
LPA receptor mRNA levels in myocardial tissue and cells. A) LPA1-6 mRNA levels in ventricles from chow and HFHS-fed mice in fed and 16-h fasted states (n = 5). B) LPA1-6 mRNA levels in cardiomyocytes isolated from chow and HFHS-fed mice (n = 3). C) LPA1-6 mRNA levels in HL-1 cardiomyocytes incubated in the presence or absence of 1.2 mM palmitate for 16 h (n = 3). D) LPA1-6 mRNA levels in atrial tissue from non-obese, pre-obese, and obese patients undergoing cardiac surgery (n = 6 for non-obese and pre-obese, n = 18 for obese). A-D) *P < 0.05, **P < 0.01, ***P < 0.001 as determined using two-way ANOVA followed by a Tukey post hoc analysis (A, D) or unpaired two-tailed t-test (B, C).

Despite recent studies implicating LPA receptor signaling in cardiovascular disease [9–13] and obesity/diabetes [20, 22–24, 30–32], it remains unclear which of the six so far identified LPA receptors (LPA1-6) are expressed in the heart and cardiomyocytes and whether myocardial LPA receptor expression is regulated by changes in metabolic status. Therefore, we examined LPA receptor mRNA levels in the heart from mice that were fed either chow or HFHS diet for 16 weeks in the fed state and following a 16-h fast. We have previously shown that HFHS-fed mice display a marked increase in body weight, impaired glucose homeostasis, and moderate cardiac dysfunction [33]. All six LPA receptors were detected in ventricles from chow and HFHS-fed mice with LPA5 exhibiting the highest mRNA expression (Fig 2A). Interestingly, mRNA levels of LPA4, LPA5, and LPA6 were drastically upregulated in HFHS-fed compared to chow-fed mice in both fed and fasted states (Fig 2A). Fasting led to a reduction in LPA5 mRNA levels, suggesting that LPA5 is not only regulated by chronic nutritional stimuli but also by acute changes in nutritional status (Fig 2A).

The heart has a diverse cellular composition that includes cardiomyocytes, endothelial cells, medullary cells [34], and fibroblasts [35]. To determine which LPA receptors are expressed specifically in cardiomyocytes, we isolated these cells from chow and HFHS-fed mice and examined the LPA receptor expression profile (Fig 2B). All six LPA receptors were detected in cardiomyocytes from chow and HFHS-fed mice with LPA4, LPA5, and LPA6 exhibiting the highest mRNA levels (Fig 2B), similar to the LPA receptor expression pattern in whole ventricles (Fig 2A). While LPA1, LPA2, and LPA3 were decreased in cardiomyocytes from HFHS-fed mice, LPA4 and LPA6 were upregulated in HFHS vs. chow-fed mice (Fig 2B), consistent with increased LPA4 and LPA6 mRNA levels in whole ventricles from HFHS-fed mice (Fig 2A). In contrast to the upregulation of LPA5 mRNA levels in the heart from HFHS-fed mice, LPA5 mRNA expression in isolated cardiomyocytes was similar between groups (Fig 2B), suggesting that non-cardiomyocyte cells contribute to the upregulation of LPA5 mRNA in the myocardium from HFHS-fed mice.

Since increased circulating lipids and ensuing cardiomyocyte lipotoxicity and insulin resistance contribute to obesity-related cardiac complications [36], we examined whether incubation of HL-1 cardiomyocytes with 1.2 mM palmitate for 16 h, which led to impaired insulin-stimulated Akt phosphorylation at Ser^473^ (S1 Fig), can recapitulate changes in LPA receptor expression detected in ventricles and cardiomyocytes from obese-insulin resistant mice (Fig 2A and 2B). Interestingly, incubation with palmitate led to the upregulation of LPA4, LPA5 and LPA6 in HL-1 cells (Fig 2C), similar to obesity-induced increases in the expression of these receptors in primary mouse cardiomyocytes and/or ventricles (Fig 2A). Interestingly, mRNA levels of LPA1 were also markedly increased in HL-1 cells exposed to palmitate when compared to control (Fig 2C). Taken together, these data suggest that all six, so far identified, LPA receptors are expressed in the murine heart and cardiomyocytes and that diet-induced obesity and acute changes in nutritional status (feeding/fasting) lead to altered expression of distinct LPA receptors, notably an increase in LPA4, LPA5, and/or LPA6, in the myocardium and cardiomyocytes.

### Patients undergoing cardiac surgery show increased LPA4 and LPA5 mRNA level in atrial tissue in proportion to obesity

To examine whether obesity-induced changes in LPA receptor mRNA levels in the murine heart also occur in humans, we determined LPA1-6 mRNA levels in atrial appendage from patients undergoing cardiac surgery who were either non-obese, pre-obese, or obese based on BMI (Tables 3–5). All six LPA receptors were expressed in the human heart (Fig 2D). Similar to the LPA receptor mRNA expression profile in mice with diet-induced obesity (Fig 2A), mRNA levels of LPA4 and LPA5 were markedly increased in atrial tissue from patients who are either pre-obese or obese compared to non-obese patients (Fig 2D). Moreover, LPA4 and LPA5 mRNA levels also significantly correlated with BMI and waist circumference (Table 6). LPA5 mRNA levels were also negatively correlated with ejection fraction (Table 6), suggesting an inverse relationship between LPA5 and cardiac function. In contrast to the increase in LPA6 mRNA levels in the obese mouse heart (Fig 2A), LPA6 expression was unchanged in atrial tissue from overweight or obese patients compared to non-obese patients (Fig 2D) and did not correlate with measures of obesity (Table 6). Similarly, LPA1, LPA2, and LPA3 mRNA levels were unchanged between groups and were not associated with BMI, waist circumference, or hip circumference (Fig 2D, Table 6). Taken together, these data suggest that all six LPA receptors are expressed in the human heart and that overweight and obesity lead to the upregulation of LPA4 and LPA5 mRNA levels in the human myocardium.

**Table 6:** Unadjusted Pearson’s correlations of LPA receptors in atrial appendage from cardiac surgery patients.

Variable		LPA1	LPA2	LPA3	LPA4	LPA5	LPA6
**Parameters of obesity**							
	**BMI**	-0.220	-0.153	-0.317	0.454	0.518	-0.247
		*0*.*242*	*0*.*418*	*0*.*087*	***0*.*012***	***0*.*003***	*0*.*188*
	**Waist circumference**	-0.269	-0.217	-0.304	0.392	0.499	-0.082
		*0*.*150*	*0*.*250*	*0*.*102*	***0*.*031***	***0*.*005***	*0*.*665*
	**Hip circumference**	-0.220	-0.274	-0.276	0.200	0.239	-0.155
		*0*.*242*	*0*.*143*	*0*.*140*	*0*.*290*	*0*.*203*	*0*.*412*
**Metabolic parameters**							
	**Triacylglycerols fasting**	0.345	0.0247	0.139	0.143	0.220	-0.209
		*0*.*062*	*0*.*897*	*0*.*465*	*0*.*451*	*0*.*242*	*0*.*267*
	**HDL cholesterol fasting**	-0.243	-0.242	-0.108	-0.027	-0.294	0.021
		*0*.*196*	*0*.*198*	*0*.*570*	*0*.*887*	*-0*.*115*	*0*.*910*
	**Cholesterol fasting**	0.082	0.037	-0.071	0.100	-0.008	0.099
		*0*.*668*	*0*.*845*	*0*.*708*	*0*.*598*	*-0*.*967*	*0*.*602*
	**LDL cholesterol fasting**	0.104	0.130	-0.087	0.051	-0.012	0.214
		*0*.*585*	*0*.*494*	*0*.*646*	*0*.*787*	*0*.*951*	*0*.*256*
	**Non-HDL cholesterol fasting**	0.194	0.140	-0.028	0.121	0.106	0.101
		*0*.*305*	*0*.*459*	*0*.*882*	*0*.*524*	*0*.*576*	*0*.*595*
	**HbA1c**	0.123	0.142	0.161	0.060	0.273	-0.310
		*0*.*516*	*0*.*454*	*0*.*395*	*0*.*753*	*0*.*143*	*0*.*095*
	**Glucose random blood**	-0.063	0.110	0.180	0.084	0.305	-0.299
		*0*.*739*	*0*.*561*	*0*.*340*	*0*.*659*	*0*.*101*	*0*.*108*
	**Fasting Glucose**	0.030	0.047	-0.034	-0.039	0.373	-0.281
		*0*.*885*	*0*.*820*	*0*.*867*	*0*.*852*	*0*.*061*	*0*.*163*
	**Fasting Insulin**	0.017	-0.111	0.166	0.014	0.052	-0.197
		*0*.*933*	*0*.*588*	*0*.*417*	*0*.*947*	*0*.*800*	*0*.*336*
	**HOMA-IR**^**2**^	0.043	-0.064	0.173	0.028	0.175	-0.263
		*0*.*833*	*0*.*755*	*0*.*399*	*0*.*892*	*0*.*391*	*0*.*193*
	**hsCRP**	-0.488	-0.331	-0.360	0.142	0.197	-0.172
		***0*.*013***	*0*.*106*	*0*.*077*	*0*.*499*	*0*.*346*	*0*.*412*
**Blood chemistry**							
	**Post-OP troponin**	-0.037	-0.283	0.043	-0.259	-0.120	-0.122
		*0*.*852*	*0*.*152*	*0*.*830*	*0*.*191*	*0*.*551*	*0*.*544*
**Echocardiography/angiography**							
	**Ejection fraction**	-0.108	-0.267	-0.028	-0.278	-0.403	-0.072
		*0*.*584*	*0*.*169*	*0*.*886*	*0*.*152*	***0*.*034***	*0*.*717*
	**Left ventricular end diastolic pressure**	0.041	0.072	-0.065	0.232	0.172	-0.015
		*0*.*838*	*0*.*722*	*0*.*748*	*0*.*244*	*0*.*392*	*0*.*940*
**NYHA Classification**		-0.242	-0.035	-0.010	0.345	0.162	-0.232
		*0*.*206*	*0*.*855*	*0*.*961*	*0*.*067*	*0*.*401*	*0*.*225*
**Post-OP complications**							
	**Length of stay**	0.003	-0.130	0.269	0.321	0.025	-0.179
		*0*.*989*	*0*.*495*	*0*.*150*	*0*.*084*	*0*.*896*	*0*.*343*

Data for each correlation are expressed as Pearson’s *r* (top) followed by *P* value (bottom, italic), denoted in bold when *P* < 0.05.

### Diet-induced obesity and insulin resistance lead to changes in the LPA receptor mRNA expression profile in murine white adipose tissue and cultured adipocytes

**Fig 3:**
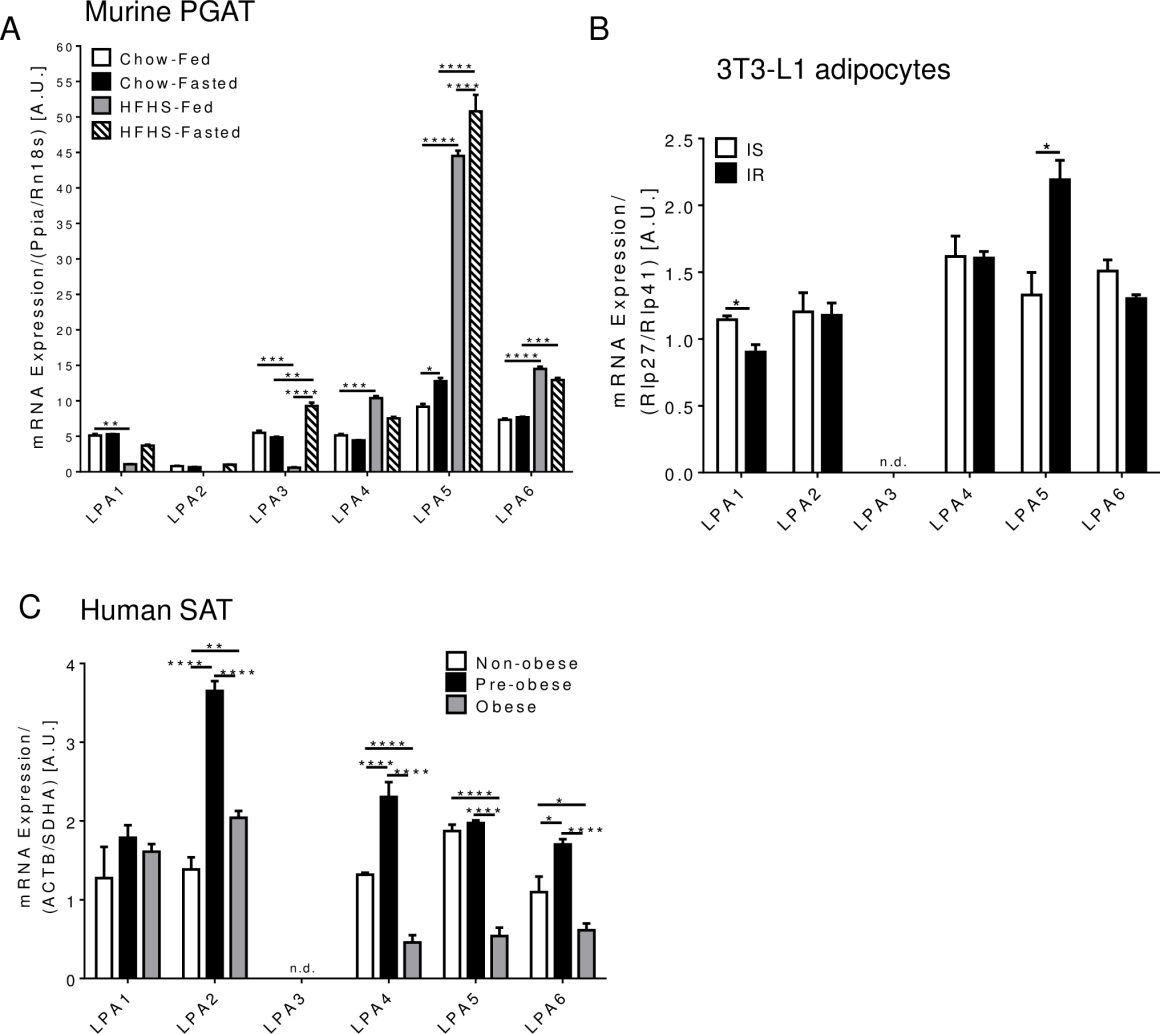
LPA receptor mRNA levels in adipose tissue and cells. A) LPA1-6 mRNA levels in perigonadal adipose tissue from chow and HFHS-fed mice in fed and 16-h fasted states (n = 5). B) LPA1-6 mRNA levels in insulin sensitive and insulin resistant 3T3-L1 adipocytes (n = 3). C) LPA1-6 mRNA levels in subcutaneous adipose tissue from non-obese, pre-obese, and obese patients undergoing cardiac surgery (n = 6 for non-obese and pre-obese, n = 18 for obese). A-C) *P< 0.05, **P < 0.01, ***P < 0.001 as determined using two-way ANOVA followed by a Tukey post hoc analysis (A, C) or unpaired two-tailed t-test (B).

Increased fat storage in adipocytes and remodeling of adipose tissue involve chronic inflammation, altered adipokine secretion and enhanced lipolysis as hallmarks of obesity. Recent studies have suggested that the autotaxin-LPA signaling pathway plays an important role in adipose tissue expansion and the development of obesity [19, 20]. However, it is incompletely understood which LPA receptors are expressed in adipose tissue and adipocytes. Therefore, we examined LPA receptor mRNA levels in perigonadal adipose tissue (PGAT) from chow and HFHS-fed mice. Our data show that all six LPA receptors are expressed in PGAT, although LPA2 was barely detectable (Fig 3A). LPA5 and LPA6 exhibited the highest mRNA expression in murine PGAT (Fig 3A). LPA1 was lower in HFHS-fed compared to chow-fed mice in the fed state while this difference was not evident in the fasted state (Fig 3A). Similarly, LPA3 mRNA levels were reduced in HFHS-fed mice in the fed state (Fig 3A). However, LPA3 was markedly upregulated in HFHS-fed mice upon fasting leading to increased LPA3 mRNA levels compared to chow-fed mice (Fig 3A). In contrast to LPA1 and LPA3, LPA4 mRNA expression was upregulated in HFHS-fed compared to chow-fed mice in the fed state (Fig 3A). LPA5 mRNA was significantly increased in HFHS-fed mice and upregulated in chow and HFHS-fed mice in the fasted state (Fig 3A).

Adipose tissue not only consists of adipocytes but also immune cells, fibroblasts, and vascular cells [37], which likely contribute to some extent to the LPA receptor mRNA levels measured in whole PGAT (Fig 3A). To determine which LPA receptors are expressed specifically in adipocytes and whether LPA receptor expression is altered under conditions mimicking obesity-insulin resistance, we incubated 3T3-L1 adipocytes with media containing high glucose (30 mM) and high insulin (100 nM) concentrations for 24 h to induce insulin resistance as previously demonstrated [24] and determined by blunted insulin-stimulated Akt phosphorylation at Ser^473^ (S1 Fig). With the exception of LPA3, all other LPA receptors were present in 3T3-L1 adipocytes (Fig 3B). Similar to the LPA1 downregulation in PGAT from mice with diet-induced obesity (Fig 3A), LPA1 mRNA levels were reduced in insulin resistant (IR) vs. insulin sensitive (IS) adipocytes (Fig 3B). Moreover, in line with a drastic increase in LPA5 mRNA levels in PGAT from obese mice (Fig 3A), LPA5 was also markedly upregulated in adipocytes following the induction of insulin resistance (Fig 3B). LPA2, LPA4, and LPA6 mRNA levels were comparable between insulin sensitive and insulin resistant adipocytes (Fig 3B). Taken together, these data suggest that obesity and insulin resistance lead to significant changes in the expression of distinct LPA receptors in murine white adipose tissue and adipocytes.

### LPA4, LPA5, and LPA6 mRNA levels in subcutaneous adipose tissue are negatively associated with markers of obesity, metabolic disease and/or outcomes in patients undergoing cardiac surgery

To examine whether obesity-related changes in LPA receptor expression in white adipose tissue are also evident in humans, we determined LPA1-6 mRNA levels in subcutaneous adipose tissue from non-obese, pre-obese, and obese patients undergoing cardiac surgery. Similar to cultured murine adipocytes (Fig 3B), LPA1, LPA2, LPA4, LPA5, and LPA6, but not LPA3 were expressed in human adipose tissue (Fig 3C). Except for LPA1, the expression of all other LPA receptors was changed either in the pre-obese and/or obese group compared to the non-obese group (Fig 3C). LPA2, LPA4, and LPA6, but not LPA5, were upregulated in pre-obese patients (Fig 3C). Interestingly, despite either increased or unchanged expression in pre-obese patients, LPA4, LPA5, and LPA6 were significantly downregulated in obese patients compared to non-obese patients (Fig 3C). Moreover, LPA4, LPA5 and LPA6 mRNA levels inversely correlated with BMI, waist circumference, and hip circumference in this patient population (Table 7). LPA5 and LPA6, but not LPA4, also negatively correlated with triacylglycerols and HbA1c, while LPA4 and LPA6 positively correlated with fasting LDL cholesterol, suggesting that LPA4, LPA5, and LPA6 are associated with glucose and/or lipid homeostasis (Table 7). Furthermore, mRNA levels of all three of these LPA receptors were negatively associated with in-hospital length-of-stay (Table 7). Taken together, these unadjusted data suggest that, in human subcutaneous adipose tissue, most LPA receptors exhibit differential expression with pre-obesity and/or obesity and that decreased LPA4, LPA5, LPA6 expression is related to an increase in certain markers of metabolic syndrome, prolonged length-of-stay post cardiac surgery, and likely predisposes to a less favourable disposition at hospital discharge.

**Table 7:** Unadjusted Pearson’s correlations of LPA receptors in subcutaneous adipose tissue from cardiac surgery patients.

Variable		LPA1	LPA2	LPA4	LPA5	LPA6
**Parameters of obesity**						
	**BMI**	0.240	0.000	-0.567	-0.747	-0.523
		*0*.*201*	*0*.*999*	***0*.*001***	***<* .*0001***	***0*.*003***
	**Waist circumference**	0.179	0.099	-0.469	-0.665	-0.474
		*0*.*343*	*0*.*601*	***0*.*009***	***<* .*0001***	***0*.*008***
	**Hip circumference**	0.102	-0.150	-0.592	-0.619	-0.481
		*0*.*591*	*0*.*428*	***0*.*001***	***0*.*000***	***0*.*007***
**Metabolic parameters**						
	**Triacylglycerols fasting**	0.032	-0.043	-0.173	-0.381	-0.366
		*0*.*868*	*0*.*823*	*0*.*359*	***0*.*038***	***0*.*046***
	**HDL cholesterol fasting**	-0.101	-0.137	0.115	0.158	0.332
		*0*.*596*	*0*.*471*	*0*.*544*	*0*.*404*	*0*.*073*
	**Cholesterol fasting**	0.143	0.010	0.258	0.165	0.269
		*0*.*450*	*0*.*959*	*0*.*168*	*0*.*383*	*0*.*150*
	**LDL cholesterol fasting**	0.139	0.061	0.369	0.339	0.385
		*0*.*464*	*0*.*749*	***0*.*045***	*0*.*067*	***0*.*036***
	**Non-HDL cholesterol fasting**	0.197	0.063	0.248	0.125	0.169
		*0*.*297*	*0*.*742*	*0*.*186*	*0*.*509*	*0*.*373*
	**HbA1c**	0.027	-0.032	-0.316	-0.494	-0.390
		*0*.*886*	*0*.*867*	*0*.*088*	***0*.*005***	***0*.*033***
	**Glucose random blood**	0.046	0.068	-0.192	-0.268	-0.201
		*0*.*808*	*0*.*720*	*0*.*310*	*0*.*151*	*0*.*286*
	**Fasting Glucose**	-0.015	0.295	0.135	-0.060	0.050
		*0*.*941*	*0*.*143*	*0*.*512*	*0*.*771*	*0*.*807*
	**Fasting Insulin**	-0.029	0.144	0.056	0.066	0.200
		*0*.*889*	*0*.*484*	*0*.*786*	*0*.*750*	*0*.*326*
	**HOMA-IR**^**2**^	-0.044	0.211	0.109	0.062	0.201
		*0*.*832*	*0*.*300*	*0*.*597*	*0*.*762*	*0*.*324*
	**hsCRP**	0.173	-0.115	-0.272	-0.061	-0.051
		*0*.*407*	*0*.*583*	*0*.*189*	*0*.*773*	*0*.*810*
**Blood chemistry**						
	**Post-OP troponin**	-0.194	-0.121	-0.242	-0.187	-0.167
		*0*.*332*	*0*.*547*	*0*.*223*	*0*.*349*	*0*.*405*
**Echocardiography/angiography**						
	**Ejection fraction**	-0.185	-0.050	-0.056	-0.013	-0.065
		*0*.*345*	*0*.*802*	*0*.*779*	*0*.*946*	*0*.*743*
	**Left ventricular end diastolic pressure**	0.033	-0.154	-0.210	-0.145	-0.142
		*0*.*871*	*0*.*442*	*0*.*293*	*0*.*470*	*0*.*481*
**NYHA Classification**		0.026	0.111	-0.218	-0.337	-0.112
		*0*.*893*	*0*.*566*	*0*.*256*	*0*.*074*	*0*.*564*
**Post-OP complications**						
	**Length of stay**	-0.100	-0.192	-0.399	-0.556	-0.388
		*0*.*598*	*0*.*308*	***0*.*029***	***0*.*001***	***0*.*034***

Data for each correlation are expressed as Pearson’s *r* (top) followed by *P* value (bottom, italic), denoted in bold when *P* < 0.05.

## Discussion

The autotaxin-LPA pathway has been linked to obesity-related insulin resistance and cardiovascular disease, which is often associated with metabolic disease, suggesting that LPA receptor signaling plays a central role in these disease states. Despite the emerging importance of LPA receptors in health and disease, little is known about the tissue expression of LPA receptors, particularly in the myocardium and adipose tissue.

In this study, we sought to comprehensively examine mRNA levels of the six known LPA receptors in heart and adipose tissue and their regulation by acute and chronic changes in metabolic status. We show that all LPA receptors are expressed in heart and adipose tissue at distinct levels, except for LPA3, which was undetectable in murine adipocytes and human subcutaneous adipose tissue. These data are consistent with a prior study demonstrating the absence of LPA3 mRNA expression in murine 3T3F442A adipocytes [38]. Obesity was associated with a marked upregulation of LPA receptors 4, 5, and 6 in the murine heart, although only LPA4 and LPA6 or LPA5 were increased in isolated murine cardiomyocytes and human heart with obesity, respectively. Moreover, LPA4 and LPA5 mRNA levels in human atrial tissue correlated with BMI and waist circumference. Obesity-induced lipotoxicity could contribute to these changes in LPA receptor expression in the heart and cardiomyocytes since incubation of HL-1 cells with palmitate also led to increased levels of LPA4, LPA5, and LPA6. While the precise relationship between lipotoxicity and LPA receptors has yet to be elucidated, it is possible that palmitate increases LPA receptor expression via pro-inflammatory transcription factors including activator protein 1 (AP-1), nuclear factor κ-light-chain-enhancer of activated B cells (NFκB), and interferon regulatory factor (IRF) [39], which were also shown to upregulate autotaxin [40–42]. LPA receptor mRNA expression in adipose tissue and adipocytes was more varied. While LPA5 was consistently upregulated in HFHS-fed mice and insulin resistant adipocytes, LPA5 mRNA was reduced in adipose tissue from obese vs. non-obese and pre-obese individuals. LPA4, LPA5, and LPA6 in subcutaneous adipose tissue were also negatively associated with measures of obesity and post-operative length-of-stay in cardiac surgery patients. Additionally, LPA5 and LPA6 inversely correlated with triglycerides and HbA1c, indicators of impaired lipid and glucose homeostasis. It remains to be determined in larger cohorts whether the relationship of LPA receptors in subcutaneous adipose tissue with metabolic parameters and outcomes post cardiac surgery are independent of obesity measures. Taken together, these data shed new light on the regulation of LPA receptors and identify specific LPA receptors that respond to acute and chronic changes in the metabolic milieu in mice and humans.

Prior studies have started to examine the regulation of LPA receptor mRNA expression and linkage to disease states, although the underlying molecular mechanisms remain largely unclear. Consistent with a critical role of LPA signaling in cancer cells, changes in LPA receptor mRNA expression have been demonstrated in a variety of cancers, including hepatocellular carcinoma, breast cancer, and ovarian cancer. For example, Enooku et al. [43] demonstrated that increased mRNA levels of LPA2 and LPA6 in hepatocellular carcinoma correspond with a higher malignant potential of the tumor, specifically poorer differentiation, microvascular invasion, and earlier recurrence in presence of elevated serum autotaxin levels. Triple receptor-negative breast cancer tissue and cells exhibited increased mRNA expression of LPA3, which was associated with tumor metastatic ability [44]. LPA2 and LPA3 mRNA levels were increased in most ovarian cancer cell lines and a substantial proportion of ovarian cancer tissues, which may contribute to ovarian cancer aggressiveness [45]. Specific receptor targeting of the LPA signaling network thus may provide novel avenues for further therapeutic development in cancer.

Besides the aberrant expression of LPA receptors in tumors, altered LPA receptor mRNA levels have also been implicated in inflammatory diseases including rheumatoid arthritis, bacterial infection, and pulmonary fibrosis. Zhao et al. [46] showed that the inflammatory cytokine, tumor necrosis factor α, stimulates LPA3 mRNA expression in human synoviocytes, which, through increased LPA-LPA3 signaling, may lead to the increased production of cytokines that play a key role in rheumatoid arthritis. Upregulation of LPA3 in a pro-inflammatory milieu was also documented in monocytes [47]. Exposure of THP-1 macrophages to lipopolysaccharide led to an increase in mRNA levels of both LPA3 receptor and autotaxin via a mechanism involving interferon-inducible double stranded RNA-dependent protein kinase and sphingosine kinase-1 [47]. Moreover, reduced lipopolysaccharide-induced CCL8 production upon LPA3 knockdown in THP-1 cells suggested that increased LPA3, along with autotaxin, contributes to inflammation following bacterial infection [47]. In pulmonary fibrosis, LPA1 mRNA appears to be markedly upregulated and LPA1 deficiency in mice resulted in reduced fibroblast recruitment and vascular leak, which may ameliorate tissue fibrosis [48]. Such advances in LPA receptor research support the development of therapeutic LPA receptor modulators. Indeed, autotaxin and LPA receptor modulators have been used successfully to treat diseases such as cancer, rheumatoid arthritis, and glaucoma in preclinical trials [49]. Furthermore, two compounds targeting LPA1 have passed Phase I and II clinical trials for the treatment of idiopathic pulmonary fibrosis, and one LPA1-3 modulator has completed Phase II trials for systemic sclerosis [49]. A target for a related lysophospholipid receptor has been FDA-approved for the treatment of relapsing forms of multiple sclerosis [50]. These advances demonstrate the promising therapeutic potential of lysophospholipid signaling modulators for chronic inflammatory disease states. Yet despite growing evidence supporting a link between LPA receptor signaling and inflammation, very little is known about the regulation of LPA receptor expression in obesity and heart disease, in which chronic pro-inflammatory signaling plays a major role.

## Conclusion

In conclusion, our study shows that mRNA expression of all six so far identified LPA receptors is highly dynamic and that LPA receptor mRNA levels in white adipose tissue and heart are differentially regulated during conditions of obesity-insulin resistance in mice and humans, in vivo and in cultured cells. These findings provide new insight into LPA receptor regulation and are expected to aid in studies examining the role of these receptors in metabolic and cardiovascular disease. Future experiments should address the precise molecular mechanisms underlying the regulation of LPA receptor expression by acute and chronic changes in metabolic status.

## Supporting information

S1 Fig Assessment of insulin resistance in HL-1 cardiomyocytes and 3T3-L1 adipocytes. A) Immunoblot and B) densitometric analysis of Akt phosphorylation at Ser^473^ in HL-1 cells incubated in the absence or presence of 1.2 mM palmitate for 18 h, followed by incubation in the presence or absence or 100 nM insulin for 15 min (n = 3). A) Immunoblot and C) densitometric analysis of Akt phosphorylation at Ser^473^ in 3T3-L1 adipocytes incubated either with high glucose-high insulin or low glucose-no insulin for 24 h, followed by incubation in the presence or absence of 20 nM insulin for 15 min (n = 3). **P < 0.01, ****P < 0.0001 as determined using two-way ANOVA followed by a Sidak’s post hoc analysis. IS, insulin sensitive; IR, insulin resistant; PS, protein stain.
(TIF)
