# Supplementary figures and images for: Lysophosphatidic acid receptor mRNA levels in heart and white adipose tissue are associated with obesity in mice and humans
Source: PLoS One. 2017 Dec 13;12(12):e0189402. doi: 10.1371/journal.pone.0189402 (PMC5728537; doi:10.1371/journal.pone.0189402)

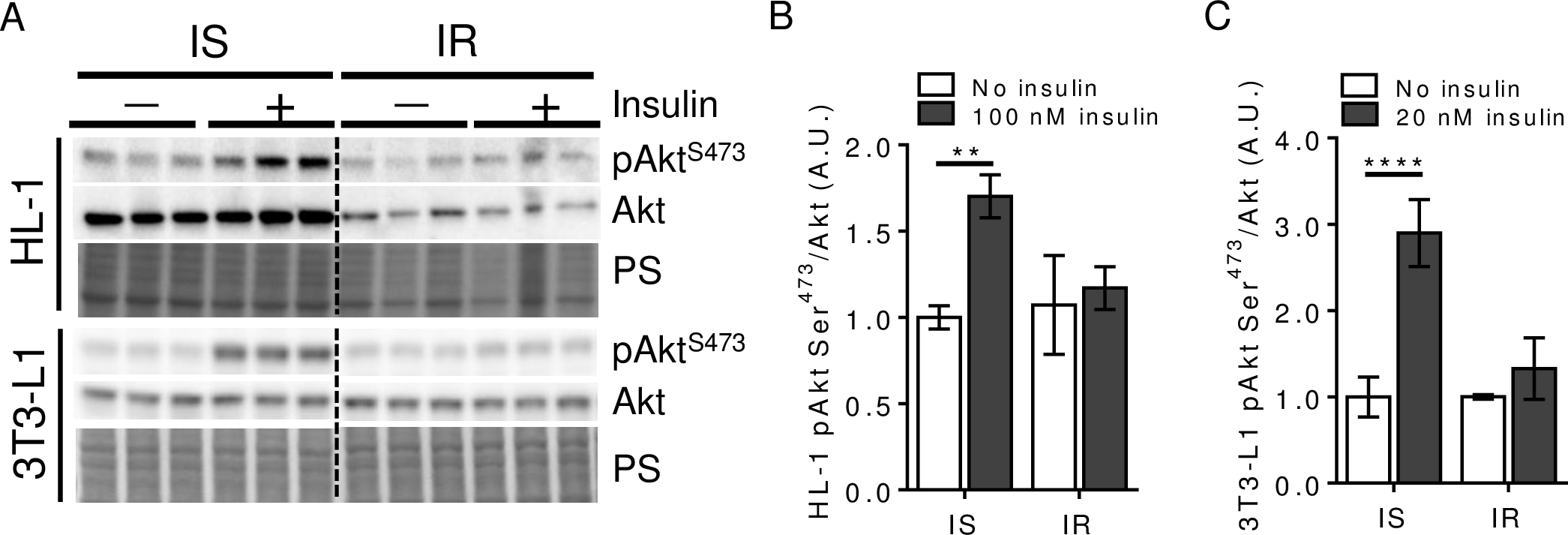

Supplement: S1 Fig — A) Immunoblot and B) densitometric analysis of Akt phosphorylation at Ser473 in HL-1 cells incubated in the absence or presence of 1.2 mM palmitate for 18 h, followed by incubation in the presence or absence or 100 nM insulin for 15 min (n = 3). A) Immunoblot and C) densitometric analysis of Akt phosphorylation at Ser473 in 3T3-L1 adipocytes incubated either with high glucose-high insulin or low glucose-no insulin for 24 h, followed by incubation in the presence or absence of 20 nM insulin for 15 min (n = 3). **P < 0.01, ****P < 0.0001 as determined using two-way ANOVA followed by a Sidak’s post hoc analysis. IS, insulin sensitive; IR, insulin resistant; PS, protein stain. (TIF) [file pone.0189402.s001.tif]
